# Mitochondrial Function as a Potential Tool for Assessing Function, Quality and Adulteration in Medicinal Herbal Teas

**DOI:** 10.3389/fphar.2021.660938

**Published:** 2021-04-26

**Authors:** Steven B. Woodley, Rhys R. Mould, Meliz Sahuri-Arisoylu, Ifigeneia Kalampouka, Anthony Booker, Jimmy D. Bell

**Affiliations:** ^1^ Research Centre for Optimal Health, School of Life Sciences, College of Liberal Arts and Sciences, University of Westminster, London, United Kingdom; ^2^ Health Innovation Ecosystem, University of Westminster, London, United Kingdom; ^3^ Research Group 'Pharmacognosy and Phytotherapy', UCL School of Pharmacy, London, United Kingdom

**Keywords:** herbal tea, herbal medicine, quality control, mitochondria, functional analysis, potency

## Abstract

Quality control has been a significant issue in herbal medicine since herbs became widely used to heal. Modern technologies have improved the methods of evaluating the quality of medicinal herbs but the methods of adulterating them have also grown in sophistication. In this paper we undertook a comprehensive literature search to identify the key analytical techniques used in the quality control of herbal medicine, reviewing their uses and limitations. We also present a new tool, based on mitochondrial profiling, that can be used to measure medicinal herbal quality. Besides being fundamental to the energy metabolism required for most cellular activities, mitochondria play a direct role in cellular signalling, apoptosis, stress responses, inflammation, cancer, ageing, and neurological function, mirroring some of the most common reasons people take herbal medicines. A fingerprint of the specific mitochondrial effects of medicinal herbs can be documented in order to assess their potential efficacy, detect adulterations that modulate these effects and determine the relative potency of batches. Furthermore, through this method it will be possible to assess whole herbs or complex formulas thus avoiding the issues inherent in identifying active ingredients which may be complex or unknown. Thus, while current analytical methods focus on determining the chemical quality of herbal medicines, including adulteration and contamination, mitochondrial functional analysis offers a new way of determining the quality of plant derived products that is more closely linked to the biological activity of a product and its potential clinical effectiveness.

## Introduction

Quality control of medicinal herbs have been an issue for as long as humans have been using plants to heal. Around 50 BCE, in Dioscorides’ *De Materia Medica*, one of the earliest written examples of a systematic pharmacopoeia, the author acknowledged the issue of adulteration though both accidental and fraudulent practices, and includes 40 examples of specific tests on how to detect them (Riddle, 1985). The majority were organoleptic, detecting adulteration through the senses, but several employed chemico-physical tests such as the ability of balsam (*Commiphora opobalsamum* (L.) Engl. *Burseraceae*) to be washed clean from a woolen cloth. A similar trend arose in Asia where the first *materia medica*, the *Shen Nong Ben Cao Jing*, written c.a. the first century CE, described the tastes, qualities and growing regions of each drug so that it could be positively identified.

Today the issue of quality control is even more pertinent, with the global herb trade being worth over US$60 billion in 2017 involving 29,000 herbal substances and growing by 15% each year (Srirama et al., 2017). Of these, herbal teas are one of the most popular methods of consuming plant materials and account for a significant proportion of this market, estimated at US$4.2 billion by 2025 (Market Research Future, 2020). This does not include the US$22.7 billion market in conventional tea (*Camellia sinensis (L.) Kuntze Theaceae*) (Market Research Future, 2019) with water-based infusions and decoctions being some of the oldest and most popular methods of extracting the medicinal properties from herbs (The Herbarium, 2009). Both of these groups will be considered under the term “medicinal herbal teas” for the purpose of this review. They differ only in that decoctions simmer the water while the herbs are being soaked and infusions are made by pouring freshly boiled water over the herbs. Infusions make up the majority of the use in the western world due to their convenience and familiarity while decoctions remain popular in Asia, although the increasing pace of life is leading many companies to develop convenient instant powders and granules from pre-decocted herbs. Alcoholic tinctures, popular among professional western herbalists for their ability to extract specific less polar active compounds (Bone, 2003) but also with a history in the east (Flaws, 1994; Sionneau, 1995), as well as pills made from raw powdered herbs or extracts, will not be considered here.

Methods for detecting adulteration have grown in sophistication as the technology to examine them has developed (Fitzgerald et al., 2020). Visual identification has been enhanced by microscopic inspection and the simple chemico-physical analyses, described by Dioscorides, has been replaced by more advanced chemometric testing in the forms of chromatography and spectroscopy developed during the twentieth century. These methods can isolate and analyse the full chemical composition of plants and prove particularly useful in detecting adulterations with drugs or contaminants that may be invisible to the human eye and undetectable to the senses of taste and smell. High performance chromatographic methods have enabled specific fingerprints of each plant’s chemical compositions, launching the development of a chromatographic atlas of herbal medicines (HPTLC Association, 2020). Recent advances in genetics have also enabled deoxyribonucleic acid (DNA) fingerprinting to be used in a similar fashion (Lou et al., 2010; Wong et al., 2018) making it possible to accurately identify a species as well as detect adulteration with plants that may look identical, even under the microscope, and with comparable chemical profiles.

Unfortunately, as the ability to detect adulteration has improved, so has the technology to adulterate herbal medicines. While much of it appears to be accidental, at the level of foraging or purchasing at a market, there is evidence to suggest that it is also deliberate, including the addition of drugs to enhance the effects of supposedly ‘natural’ supplements (Booker et al., 2016), dyes that make the color of vibrant herbs look more potent (Müller-Maatsch et al., 2016; Booker et al., 2018) and the substitution of herbal material which may have comparable chemical profiles and so evade detection by all but the most sophisticated tests (Booker et al., 2018; Frommenwiler et al., 2019).

Mitochondria stand at the center of a plethora of cellular activities, including controlling energy metabolism, the production of reactive oxygen species (ROS), apoptosis, cell division, immune signaling, and even at the very evolution of complex multicellular life (Lane, 2018). This suggests that every bioactive substance will have some effect on mitochondrial function. Many medicinal herbal teas are marketed in an overly simplistic manner for their antioxidant benefits (Paur et al., 2011) although it remains unproven and unclear whether antioxidant consumption improves health outcomes *in vivo* (Berger et al., 2012). However, the fact that herbal and traditional plant medicines have some of the highest levels of antioxidants of any foodstuffs (Carlsen et al., 2010) suggest that many of their purported therapeutic properties would involve the regulation of mitochondrial systems. We suggest that by profiling these effects, it may be possible to develop a library of mitochondrial fingerprints for individual herbs. This library, adjunct to those that exist for high performance thin layer chromatography (HPTLC) and DNA profiling databases, would be a powerful tool, not only to understand herbal function, but also determine adulteration and potency, something currently lacking in standard tests.

The great advantage of measuring biological activity directly, rather than by analysing composition, is that the aim of medicinal herbs is to modulate biological systems and in some cases, this may not be due to the presence of any single active ingredient. Chemical and DNA profiles may therefore be misleading, especially when there are multi-herb blends as commonly observed with commercial teas and medicinal formulas. In contrast, if the mitochondrial action of a formula can be mapped, in conjunction with chemical and DNA profiles, then it will be possible to compare batches and formulations against standardised mitochondrial fingerprints. Once a unique pattern has been detailed, the potency of each batch can be ascertained.

## Methods

This study was conducted in accordance with PRISMA guidelines for systematic reviews.

### Search Strategy

We searched PubMed and Google Scholar for literature on the existing methods of examining herbal teas for quality control. The search terms used were:

*PubMed*: ((chromatography) OR (spectrometry) OR (spectroscopy) OR (NMR) OR (ultraviolet) OR (infrared) OR ((DNA) OR (genetic) barcoding) OR (bioassay)) AND ((herbal tea) OR (tisane) OR (decoction)) AND ((adulteration) OR (quality) OR (contamination)). With the filters “Full Text” and “English” language applied, obtaining 363 results.

*Google Scholar*: allintitle: "herbal tea" (chromatography OR HPLC OR HPTLC OR GC OR spectrometry OR spectroscopy OR MS OR NMR OR ultraviolet OR infrared OR "DNA barcoding" OR "genetic barcoding" OR bioassay) obtaining 41 results. Six duplicates were removed before screening commenced.

### Inclusion and Exclusion Criteria

Exclusion criteria for screening was that publications must contain author, title, date of publication, abstract and that the full text was available in English. Inclusion criteria for eligibility was that the publications were peer reviewed papers, investigating teas, decoctions or water extractions and investigating quality control.

## Results

### Included Studies

**FIGURE 1:**
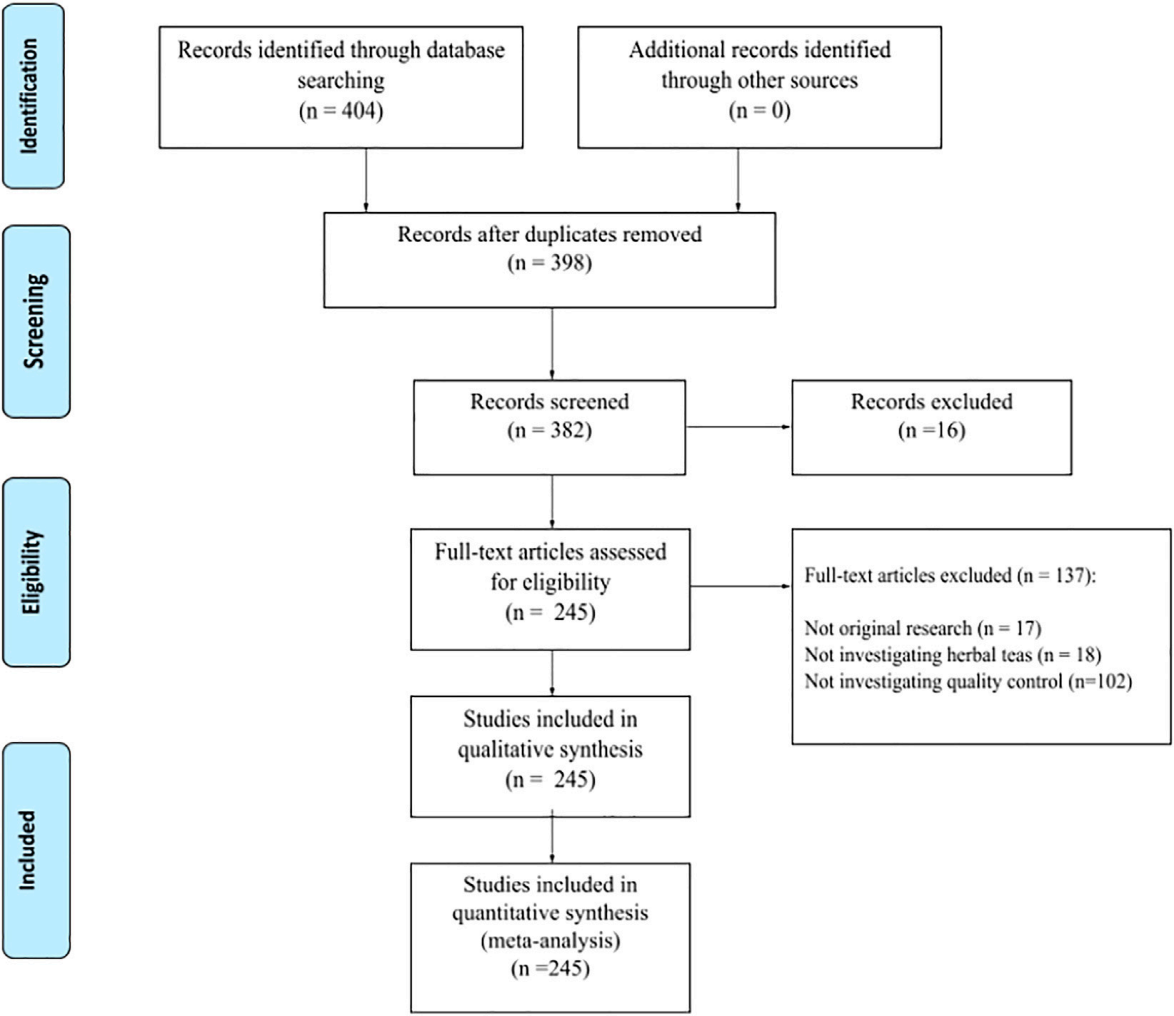
PRISMA flow diagram of the literature review process. Adapted from Moher et al (2009).

A total of 398 papers were initially identified after a preliminary search of the databases, 245 papers were included in the final analysis, with a total of 153 excluded based on the above described criteria (Figure 1). A complete table of all included papers, their methods and objectives can be found in the Supplementary Information (Table S1).

By providing a breakdown of the methods and their application (Table 1) it can be seen that chromatography is the most popular technique, with liquid chromatography (LC) being the most favored method overall, followed by mass spectrometry (MS). This is mainly because both of these techniques can be used to separate as well as analyse herbal extracts, which is an essential endpoint when assessing complex mixtures as normally found in herbal tea blends. Each individual method shall now be described with its advantages and disadvantages, including a summary of what the literature revealed.

**TABLE 1:** Occurrence of methods used in quality control of herbal teas

Method	Number of studies	Percentage
Liquid chromatography (LC)	178	72%
Gas chromatography (GC)	18	7%
Thin layer chromatography (TLC)	11	4%
Mass spectrometry (MS)	129	53%
Nuclear magnetic resonance (NMR)	13	5%
Ultraviolet-visible spectroscopy (UV-Vis)	57	23%
Infrared spectroscopy (IR)	8	3%
Optical emission spectroscopy (OES)	4	2%
DNA barcoding	15	6%
Biological assays	31	13%

### Chromatography

The literature review revealed chromatographic methods as the most popular for identifying adulteration of herbs. This is due to the fact that they can separate complex compounds into their unique components creating a ‘chemical fingerprint’ which can be used qualitatively and quantitatively (Hansen, 2015). All chromatographic methods achieve this through the universal principle of separating mixtures by distributing its components between two phases: a mobile phase, which carries the components through a medium, and a stationary phase that remains fixed causing the various constituents to separate as they migrate at different speeds (Liu, 2011). The chemical fingerprint that arises can then be compared against a reference standard or used as a preparatory step toward further analysis of the individual compounds using spectroscopic methods to identify unknown compounds. The combination of a separation technique and one or more spectroscopic detection methods is known as a hyphenated technique with almost every published chromatographic study utilising at least one spectroscopic technique. This reveals one of the main limitations of all chromatographic methods, as well as their strength, for while its analytical capacities are limited to observation of analyte peaks, it excels in separating individual components which can act as an initial preparatory step to further analysis (Braithwaite and Smith, 1999).

There are many different types of chromatographic techniques, usually named after one of their phases or their method of interaction. Of the types used in the analysis of herbal teas, liquid and gas chromatography (GC) are named after the mobile phase while thin layer chromatography (TLC) refers to the type of stationary phase used (Coskun, 2016). Of particular interest are “high performance” varieties of LC and TLC which use automated techniques to provide more accurate, reproducible readings. Each technique has been shown to have its own advantages and disadvantages.

### Liquid Chromatography (LC)

LC is one the most popular techniques found in the literature with a total of 177 papers included in the review. While LC simply refers to the state of the mobile phase, the majority of papers analysing herbs used “High Performance Liquid Chromatography” (HPLC) or “Ultra-High Performance Liquid Chromatography” (UPLC). Here the liquid mobile phase is pumped through a solid adsorbent stationary phase in a column under high pressure enabling sufficient resolution to be used quantitatively, while LC is primarily used as a preparative technique for other forms of analysis. The main difference between the HPLC and UPLC is the relative pressure used and the resolution achieved, with the latter having superior resolution and speed (Dyad Labs, 2018). HPLC and UPLC account for 150 of the 178 LC papers (84%), with only 35 (20%) using the standard form (7 using both). These methods are especially useful for quality control because of the process being largely automated which makes the results highly reproducible. The results are usually presented as a line graph where the peaks can be compared to show the presence of various compounds and their relative quantity making it ideal for generating a chemical ‘fingerprint’ against which other samples can be compared. This is reflected in the overwhelming majority of the papers using LC to identify quality markers in order to standardise market products.

Table 2 lists the uses of all forms of LC in the analysis of herbal teas included in the review. The primary use of LC is to separate out compounds for further analysis with spectroscopic techniques but HPLC features ultraviolet detection as an integral part of the system to detect when different compounds are eluting from the column. However, 106 (60%) of the studies included in the review using HPLC also hyphenated it with another spectrometric or spectroscopic technique such as MS, nuclear magnetic resonance spectroscopy (NMR) or infrared spectroscopy (IR). MS was the most popular with 102 papers combining it with LC while only eight used NMR and six of those used it in conjunction with MS. Only two papers used IR, neither in conjunction with any other technique.

**TABLE 2:** Uses of liquid chromatography in the analysis of herbal teas

Uses of liquid chromatography	Number of studies
To identify quality markers	132
To detect mycotoxins	11
To detect pyrrolizidine alkaloids	10
To detect pesticide residues	8
To detect adulterant species	7
To detect polycyclic aromatic hydrocarbons	3
To detect adulterant drugs	3
To detect tropane alkaloids	3
To detect heavy metals	2

LC appears to be the primary choice in the analysis of herbal teas since they are naturally delivered in liquid form. It therefore does not require the samples to be subjected to additional processing, preserving less stable compounds. However, it is less suited in the analysis of volatile compounds for which GC is the technique of choice, provided that they are unchanged by heat (Ng, 2017). LC is especially useful for analysing samples containing salts or carrying a charge which cannot be analysed with GC (Painter, 2018).

These limitations are illustrated by Kokalj et al. (2014) who claimed to provide the first detailed report of the chemical composition of *Tiliae flos* (*Tilia cordata* Miller, *T. platyphyllos* Scop. and *T. x vulgaris* Heyne, *Tiliaceae*) based on their procyanidin content. However, their analysis disregarded the volatile content, despite acknowledging that it was also important for quality control. Yap et al. (2007) also encountered a similar problem while attempting to develop a method of quality control for *Dang Gui Bu Xue Tang* (a combination of *Astragalus membranaceus* (Fisch.) Bge., *Fabaceae* and *Angelica sinensis* (oliv.) Diels, *Apiaceae*), reporting difficulties in detecting the components carvacrol and Z-butylidenephthalide due to their volatility, despite the volatile oils of *A. sinensis* being important for many of its bioactive effects.

### Gas Chromatography (GC)

GC works along the same principles as LC except that the sample is vaporised and carried by an inert gas (the mobile phase) into the stationary phase, under controlled temperature (Turner, 2020). The stationary phase is usually a liquid coated on a solid support contained within a glass column which elutes the components of the mobile phase at different rates, depending on their different interactions with the stationary phase. The resulting extraction is then passed to a detector which displays the results as a chromatogram. One of the great advantages of GC is the ease with which it can be connected to MS by using a mass analyser as the detector. Of the 18 studies included in the review that used GC, 15 (83%) were associated with MS.

One of the most notable trends that emerged from the GC literature was its use in detecting pesticides (Table 3). Seven out of 12 papers (58%) that searched for pesticides used GC, with a relatively high number detecting polycyclic aromatic hydrocarbons (2 out of 5, the other 3 using LC). This popularity is because it can be both selective and sensitive with simultaneous detection of many residues at lower concentrations compared with other techniques (van der Hoff and van Zoonen, 1999) giving it an important specialist role in the quality control of herbal teas, especially in the analysis of volatile compounds, including many environmental pollutants. However, it is not well suited to analysing any compounds which are affected by heat, contain salts or carry a charge for which LC is the technique of choice (Ng, 2017; Painter, 2018).

**TABLE 3:** Uses of gas chromatography in the analysis of herbal teas

Uses of gas chromatography	Number of studies
To identify quality markers	7
To detect pesticide residues	7
To detect heavy metals	3
To detect polycyclic aromatic hydrocarbons	2
To detect adulterant drugs	1

One interesting use of GC was in providing a chemometric revision of the aromatic sensory descriptions of honeybush (*Cyclopia* spp. Vent., *Fabaceae*) and rooibos (*Aspalathus linearis* (Burm.f.) R. Dahlgren, *Fabaceae*) tea traditionally used to assess quality (Du Preez et al., 2019). While the authors’ claim that this improves our understanding of aroma descriptions is true, it could also be used to artificially modulate the scent of low quality herbs.

### Thin Layer Chromatography (TLC)

TLC uses a thin layer of material made of small particles for the stationary phase. This causes the mobile phase to rise along the surface via capillary action at a constant rate and the compounds adhere to the vacant spaces (termed adsorption) at differing rates depending on their relative solubility in the mobile phase and their affinity for the stationary phase. These then remain in place after the mobile phase has evaporated and can be derivatized by various treatments to make all the analytes detectible (CAMAG, 2020). It is a less popular method of analysing herbal teas than LC with only 11 papers included in the review utilising this method but deserves a thorough examination because its use for analysing herbal materials is expanding.

Two thirds (7) of the papers included in the review used TLC to find identifying quality markers to confirm a correct species; of the others, the uses were as varied as detecting adulterant species (Shen et al., 2012; Lam et al., 2016; Guzelmeric et al., 2017), adulterant drugs (Miller and Stripp, 2007) or moulds and mycotoxins (Halt, 1998). These show the potential for TLC to be almost as varied as for LC.

As can be seen from these papers, a high performance variety of TLC has been developed similar to the high performance version of LC. It is a relatively new technique evidenced by the fact that far fewer papers have been written using it, with only five included in the present search, all being written in the last 8 years. However, its high throughput speed, low cost and options for analysing the results visually or by computer readout means that it is rapidly becoming the technique of choice for verifying species and detecting adulteration in herbal medicines (Omicron, 2018; Frommenwiler et al., 2019). The number of studies found in the present search belies the popularity and potential of HPTLC in herbal medicines research due the fact that it was restricted to teas while most studies investigate supplements and raw herbs using other solvents. If the search is modified to only “(HPTLC) and (herbal medicine) and [(adulteration) or (quality) or (contamination)]” then 45 results are returned. There is no reason why HPTLC cannot be used with water extractions and Guzelmeric et al. (2017) even found that it surpassed HPLC in detecting species adulteration of chamomile (*Matricaria chamomilla* L., *Asteraceae*) herbal teas, so the literature using this method is certain to expand.

One of the reasons for HPTLC becoming the technique of choice for analysing herbal medicines is that the results can be readily displayed as the mobile phase travels up the plate which in turn can be easily compared to reference standards or adulteration markers. This also facilitates a high throughput with faster analysis than HPTLC due to being able to perform several analyses in parallel (Bairy, 2015) and the small size of the mobile phase means that it is relatively inexpensive on materials (Loescher et al., 2014). This has led to a rapid growth of an HPTLC atlas of plants (HPTLC Association, 2020) which is being used as a reference compendium for all herbal products. One limitation of HPTLC is that it is predominantly a qualitative or semi-quantitative technique, however, as with other chromatography methods, it can be linked to quantitative analytical hardware, e.g. MS, where higher precision is desired.

### Spectroscopy/Spectrometry

Spectroscopy is the study of how radiated energy and matter interact; spectrometry is the measurement of this interaction (ATA Scientific, 2020). Some form of energy is projected at the matter which absorbs it creating an excited state. This then generates some form of electromagnetic waves which can be observed and measured.

### Mass Spectrometry (MS)

Through the use of mass-to-charge ratio (m/Q) of ions MS can reveal the elemental or isotopic signature of a molecule or compound and enable its quantification. First, the substance under investigation is ionised in order to make it susceptible to influence by a magnetic field. Then, the charged ions are accelerated to a known speed and deflected using a magnetic field (Clark, 2019). The degree to which the magnetic field deflects the ionised molecules from their course is assessed and used to determine its molecular weight. This process can be further refined by adding an additional mass analyser, known as tandem mass spectrometry, MS/MS or MS^2^, (Mittal, 2015). “Time of flight” (TOF) is one of the most common forms of tandem mass spectrometry based on the principle that heavier ions travel more slowly than lighter ions (Gilhaus, 2005). Another is the use of four cylindrical rods (a quadrupole) that generate oscillating electrical fields selecting specific ions based on the stability of their trajectories as they pass through. Quadrupoles are often set up in a triplicate formation, called a “triple quadrupole” (QqQ), where the first quadrupole is used to select certain ions, the central quadrupole acts as a collision chamber to fragment them and the last selects the fragments to be analysed (Schreiber, 2017). These setups are commonly used in biomolecular research of complex molecules.

MS is by far the most popular spectroscopic method utilised in the analysis of herbal teas. A total of 129 papers included in the review using some form of MS were identified. This is probably due to its ability to identify and characterise unknown compounds, which gives it an enormous advantage over chromatographic techniques when analysing newly identified plant compounds or forensically analysing adulteration of herbal preparations. However, 113 (88%) of these studies combined MS with some form of chromatography in order to separate the compounds before analysis, with LC being the most common (102, or 90%), followed by GC (15, or 13%) and less frequently with TLC (3, or 3%). This preparatory step can have an enormous influence on the results as can be seen by the MS studies searching for mycotoxins in tea (*C. sinensis*). Reinholds et al. (2020) found that in 140 different samples almost 97% of black teas, 88% of green teas and 100% of oolongs included in their study contained quantifiable levels of fungus with all Puerh (Chinese fermented tea) samples containing mycotoxins despite having the lowest levels of fungal contamination. Conversely, Monbaliu et al. (2010) analysed 91 teas and found only one sample of Ceylon melange to be contaminated with no mycotoxins in the drinkable products despite also testing Puerh. This is significant as Puerh is a fermented variety known to contain high concentrations of mould (Sedova et al., 2018). Both used UPLC systems to prepare the samples so unless their storage conditions prior to analysis were dramatically different, these two studies show that despite the detection capabilities of MS, the results can be easily swayed by even small variations in the preparatory steps.

From the distribution of uses for MS (Table 4) we can see a similar spread of uses to LC where the main usage is to identify and characterise marker compounds for future quality control. One of the main differences is that many of these studies are characterising a plant and identifying markers for the first time, or where a forensic analysis of herbs was being undertaken without prior knowledge of what the researchers were searching for. An important consideration when using MS, despite its outstanding sensitivity, is its relatively high cost, approximately US$500,000 capital cost, with US$250,000 yearly maintenance fees (El-Khoury, 2018) making them the domain of research laboratories or larger QC departments.

**TABLE 4:** Uses of mass spectrometry in the analysis of herbal teas

Uses of mass spectrometry	Number of studies
To identify compounds as quality markers	81
To detect pyrrolizidine alkaloids	13
To detect heavy metals	10
To detect adulterant species	8
To detect mycotoxins	8
To detect pesticide residues	5
To detect adulterant drugs	2
To detect polycyclic aromatic hydrocarbons	2

### Nuclear Magnetic Resonance Spectroscopy (NMR)

NMR is one of the preeminent techniques for determining the structure of organic compounds (Aryal, 2018). By placing molecules in a strong magnetic field and exciting its basic nuclear constituents (eg ^1^H, ^13^C, ^31^P), structural and quantitative information can be readily obtained (Emery Pharma, 2018; Zinkel, 2019).

Although NMR is renowned in its capability to determine the molecular structure of any unknown organic compound, it suffers from some considerable limitations in relation to quantitative analysis. These include its relative insensitivity requiring 10–100 times the concentration of sample (>1 μM) compared to MS, LC or GC (10–100 nM), making it unsuitable for detection of trace metabolites (Emwas et al., 2019). Another drawback is the expense of the equipment with initial capital costs often exceeding US$1 million, although smaller, lower resolution benchtop versions are available for under US$100,000 (Reisch, 2015). These limitations are reflected in the literature with only 13 papers utilising NMR with almost half of these (6) also using MS and often adding NMR to acquire additional information when MS was not specific enough. Interestingly, only one study explored the idea of using high resolution ^1^H NMR spectroscopy with multivariate statistical analysis as a method to identify the composition of complex mixtures of herbs without the use of prior separative techniques (Marchetti et al., 2020) building on the work of Booker et al. (2014) who used the same technique to analyse chemical variability of turmeric (*Curcuma longa* L. *Zingiberaceae*) along value chains. This highlights one of its considerable advantages, to be able to provide a herbal ‘fingerprint’ for a wide range of compounds, of differing polarity, without the need to separate them using chromatographic methods. Given the relative expense generally involved with NMR, this technique is an unlikely method to be routinely used in the herbal tea industry. However, once the equipment is available, sample running costs are comparatively low and so there are some opportunities for contract analysis e.g. through universities.

### Ultraviolet-Visible Spectrophotometry (UV–Vis)

Works by passing wavelengths of light in the ultraviolet (100–400 nm) and visible (400–700 nm) regions of the spectrum through a sample and measuring the light transmitted on the other side (Raja and Barron, 2020). The wavelengths not transmitted indicate the photons that have exactly matched the energy band gap required to promote a molecule from its ground state to an excited state have been absorbed. This provides an absorbance spectrum readout that can be compared to published literature for qualitative identification or quantitatively assessed, measuring the concentration of a sample using Beer’s Law if the absorptivity of a substance is known, or can be calculated (Venton, 2017). The light source is usually a deuterium lamp that emits light in the 170–375 nm ultraviolet spectrum, and a tungsten filament lamp, which produces light from 350 to 2,500 nm for the visible range, which is then filtered for specific wavelengths. Alternatively a diode-array which can measure a whole spectrum of light in a single run can also be used (Dong and Wysocki, 2019).

Of the 58 studies included in the current review, 56 (97%) coupled UV-Vis with HPLC. This is a common combination and an integral addition to HPLC kits (Cross, 2016) meaning that the other HPLC studies will have also used UV-Vis but did not discuss the results in their papers.

The reliance of UV-Vis on published literature demonstrates one of its major limitations. While most of the studies (55 or 95%) looked at assessing herbal medicines by comparing their analytes against known constituents, Zhao et al. (2012) proposed UV-Vis as a method of detecting adulteration of Chinese medicines with drugs. While they were able to successfully identify 11 antihypertensive drugs that had been mixed into herbal medicines, their only samples were preparations that they had spiked themselves and therefore had the references to hand. When herbal medicines are adulterated with drugs, they are increasingly using designer analogues in order to avoid detection making techniques such as LC-MS and NMR necessary for their identification (Haneef et al., 2013; Patel et al., 2014).

### Infrared Spectroscopy (IR)

IR, similarly to UV-Vis utilises specific wavelengths of radiation. The infrared light interacts with the bonds of molecules causing them to stretch or bend in symmetric or asymmetric ways when energy is absorbed from a very particular wavelength which in turn will depend on the functional groups within the molecule (Reusch, 2013). Troughs in transmittance in the region above 6.5 µm gives information about functional groups, while the region below 6.5 µm is known as the fingerprint region and gives a very intricate pattern that can be used to determine the chemical composition of compounds. IR can take place in the near-, mid- or far-infrared regions, or use a Fourier Transform method to simultaneously beam many frequencies, repeated in bursts of different combinations over a short time and then calculate the absorbance at each wavelength. The near range, from 780–2,526 nm is the most commonly used in quality control due to having higher energy and penetration capacity and producing less heat (Zeng et al., 2011) while the mid-infrared spectrum (2.5–25 µm) is superior for identifying structure and functional groups (Liang et al., 2011).

A total of eight papers included in the review used IR despite its versatility. Kokalj et al. (2014) found it to be a suitable low cost, rapid and simple method of quality control for both single herbs and mixtures in routinely tested samples. Ma et al. (2017) found IR could successfully determine adulteration of Chinese yam (*Dioscorea polystachya* Turcz., *Dioscoreaceae*) powder with cheaper corn and wheat starches while Yap et al. (2007) found it capable of differentiating Asian from American ginseng (*Panax ginseng* C.A. Meyer and *P. quinquefolius* L., *Araliaceae*). Lee et al. (2014) found IR outperformed GC when identifying caffeine and catechin content of tea (*C. sinensis*), while Chen et al. (2019) found IR to be of equal effectiveness to HPLC in evaluating the quality of *Rhizoma Atractylodis* (*Atractylodes macrocephala* DC., *Asteraceae*) decoction pieces. Cebi et al. (2017) also found IR could detect sibutramine in tea and coffee samples, although like UV-Vis, only spiked samples were used and analogues may be more problematic. Despite the broad range of applications which would seem to suit small herbal tea companies in Europe, the literature is largely dominated by MS and NMR data from universities, pharmaceutical corporations and the Asian herbal medicine market which is often government backed and far more integrated into conventional healthcare than in Europe or the US (Liu and Salmon, 2010). These organisations already have access to MS and NMR equipment and most smaller labs have UV-Vis fitted as standard equipment on their chromatography devices. This means an extra expense to install an IR device, train staff in its use and to develop the reference models for which there are few available in English and whose development requires time and expertise in chemometrics resulting in its low popularity as a method of analysing herbal teas (Zeng et al., 2011).

### Optical Emission Spectroscopy

This is one of the less common forms of spectroscopy used in the quality evaluation of herbal teas, only appearing in five papers but worth mentioning because it has a very specific application for detecting metal content. This is because it functions by generating a spark between an electrode and a metal sample while in a high energy inductively coupled plasma state and using a spectroscope to detect the unique spectrum specific to each element (Shimadzu, 2020). All five papers utilising this method were using it to evaluate potentially toxic levels of metals in herbal teas with Malik et al. (2013) and Rubio et al. (2012) finding levels of aluminum high enough to warrant imposing consumption limits in hibiscus (*Hibiscus sabdariffa* L., *Malvaceae*) and mint (*Mentha sp.* L., *Lamiaceae*) respectively.

### Biological Methods

Biological methods differ from those above in that they use biological systems to test for authenticity and quality in herbs. The most widespread method is certainly genetic testing but there are also several examples utilising biological assays to assess the effects of herbs and extracts on cell cultures and even *in vivo* preclinical models.

### DNA Barcoding and Genetic Analysis

DNA barcoding takes a small section of genetic code from an unidentified organism and compares it to a reference library of DNA sections such as the Barcode of Life Data (BOLD) systems database (iBOL, 2020). It is often quicker and more precise than traditional taxonomic classification but the reliability of data is only as good as the database and, in the rush to categorise as many species as possible, there are many errors that may be present (Lathe et al., 2008). Countries, where there is a strong vested interest in herbal medicine, have created large herbal medicine genetic databases such as the Medicinal Materials DNA Barcode database of Traditional Chinese Medicines (Lou et al., 2010; Wong et al., 2018) but where there is less of a vested interest in employing curators to error check and correct herbal plant materials, the number of vouchered species is likely to be less and the database of poorer quality.

Fifteen studies included in the review used genetic based methods. As expected, all were being used to identify the correct species, half (7) to check for adulterants and identify them, the rest to confirm the species when using another technique for identification. Some of these papers provide a disquieting glance of how frequently tea adulteration takes place in the commercial arena (Table 5).

**TABLE 5:** Results of genetic analysis on teas searching for adulteration.

Author	Aim	Result
Osathanunkul (2018)	Find adulterants in Soursop (*Annona muricata* L. *Annonaceae*) teas	Three out of eleven (27%) samples contained incorrect species
Omelchenko et al. (2019)	Examine 6 herbal teas, 6 herbal medicines & 6 spices for adulteration	Twelve (67%) products contained different materials to those labelled. 6 likely to be economically motivated
De Castro et al. (2017)	Examine 32 herbal teas	Two (6%) found to be adulterated
Olivar et al. (2016)	5 *Vitex negundo* L. *Lamiaceae* samples, often used as herbal tea in the Philippines	Only one satisfied the database criteria for genetic authenticity
Xin et al. (2015)	Authenticate 90 commercial *Rhodiola crenulata* (Hook.f. & Thomson) H. Ohba *Crassulaceae* products from hospitals and drug stores	Only 36 (40%) contained the correct species. 35 (38.9%) contained *R. serrata* H. Ohba and 9 (10%) *R. rosea* L. Remaining 10 (11.1%) were 3 other R. species
Duan et al. (2017)	Identify species in *Radix Clerodendrum* tea samples used in the Dai ethnic group’s medicine	Of 27 samples, only 1 (3.7%) was authentic *Clerodendrum japonicum* (Thunb.) *Lamiaceae*. Most were another medicinal species but 4 were potentially toxic *Lantana camara* L. *Verbenaceae*
Wang et al. (2016)	To find a DNA signature region which can be used to identify *Angelica sinensis* (Oliv.) Diels. *Apiaceae* in decoction powders	Of 9 decoction powders, 7 (78%) were identified as *Angelica pubescens* Maxim. *Apiaceae*

One of the complications in the use of DNA barcoding in the taxonomy of traditional medicines is that these methods were not utilised by the original practitioners when describing the original materials. Therefore a number of species can fit their description and we are now forcing their morphological and ecological descriptions to fit a narrow DNA profile. This is evident in the way that many traditional medical books have multiple species assigned a single entry (Bensky et al., 2004). Hence, when analysing the medicine of a traditional ethnic group such as Duan et al. (2017) undertook of the Dai people’s medicinal plant Ha-Bin-Liang and found that only one of the 27 samples matched *Clerodendrum japonicum* (Thunb.) Sweet, *Lamiaceae* listed as the correct species in the pharmacopoeia, they assume a priority position of DNA barcoding over the practitioners who defined the medicinal material in the first place.

### Bioassays

The use of bioassays presents an interesting opportunity for determining quality of herbs by directly measuring their effects on a biological system. It could be argued that this is the most crucial factor in any form of quality control, since functionality is at the core of herbal teas and medicinal plant consumption. With most herbs the identity of the component(s) responsible for a given effect are often uncertain or completely unknown. Moreover, given that it is recognised by herbal practitioners that rare, unavailable or ethically suspect herbs can be substituted with a taxonomically distinct species that has comparable effects (Selvam, 2010; Fang et al., 2013; Hall, 2019), measuring the effect of a herb or blend directly should be central in assessing quality control.

A total of 29 papers included in this review were identified as using some form of biological assay to assess the quality of herbal teas. With the exception of Wu et al. (2018), the reported biological assays were accompanied by a variety of adjunct methodologies to examine the chemical composition of the herbs. A plethora of assays were used for different purposes, some of which are outlined in Supplementary Table S2.

The most common assay used was the 2,2-diphenyl-1-picryl-hydrazyl-hydrate (DPPH) antioxidant assay, employed by 12 of the 29 papers (41%) included in this review, followed by the Ferric Reducing Antioxidant Power (FRAP) assay (5 papers, 17%) and various assays to measure the total phenolic content (4 papers, 14%). Since oxidative stress is a byproduct of mitochondrial activity, these studies suggest that mitochondrial function plays an important role in determining the functional quality of herbs. Despite this none of the studies looked at mitochondria directly.

Bioassays tend to be used to find a biological mechanism of action behind specific compounds which can then be selected for quality control and measured with chromatographic or spectroscopic methods. They are rarely a method of quality control in themselves. This reveals a distinct pharmaceutical bias in the fields of quality control of herbal teas, where the search for a ‘single active ingredient’ has become the main driving force in basic research, despite the fact that the use of isolated active compounds frequently leads to ineffective or less effective outcomes (eg. Lu et al., 2011). Of late, this has led modern drug discovery to look at synergies between multiple compounds and complex effects using -omics analysis (Thomford et al., 2018).

### Mitochondria in Quality Control of Medicinal Herbal Teas

The existing methods of quality control in herbal teas focus heavily on chemometric testing for specific marker compounds. When there is attention to biological activity, it is largely to test novel compounds in order to determine which ones are biologically active and can potentially be developed into drugs. These often become the primary quality markers (eg., Qin et al., 2019), followed by compounds unique to the species and then specific levels of others in relation to other species. This reveals a distinct tendency toward the pharmaceuticalisation of herbal medicine and represents a problem when dealing with complex mixtures.

One area that remains problematic for detection of herbal adulteration is the practice of mixing old herbs that may be losing their potency, with small amounts of fresh herbs. These ‘mixtures’ will share the same relative chemical and DNA profile, albeit with some reduction in certain active ingredients and only a fully quantitative methodology would be able to detect any potential ‘adulteration’.

One approach to overcome this issue would be a method that tests a herbal blend functionally, based on what it does to a complex biological system when viewed as a whole rather than the sum of its parts. Several attempts to use bioassays to ascertain the effectiveness of herbs on their intended condition have the potential to achieve this. For example, Shen et al. (2008) used a 48/80-induced histamine test on rat peritoneal cells to assess which species of magnolia flowers (*Magnolia* L. *Magnoliaceae*) should be considered the medicinal variety for rhinitis and Wu et al. (2019) used a xylene-induced ear oedema model in mice to determine the best *Polygonum chinense* L., *Polygonaceae* species to use for its anti-inflammatory effects in Liang Cha (Chinese cool tea). If a batch of herbs could reproduce these activities it may be considered functionally correct. However, in both of these cases animals had to be sacrificed making these methods unethical for routine testing in herbal quality control and most suitable for determining the best species which are then identified chemometrically. Currently there is no universal tool that can directly profile the biological effects of herbal medicines on the body.

Functional mitochondrial testing presents a unique opportunity to tackle this issue as these organelles are integral to most biological functions, providing the energy necessary to perform most tasks as well as generating important signaling mechanisms both within (Quirós et al. (2016) and between cells (Liu and Ho, 2018; Picard et al., 2018) through DNA, redox, hormonal, neurological and immune mediated pathways.

Here we discuss the potential of using a plethora of mitochondrial functions as the basis for a functional pipeline for the testing of herbal teas. As well as describing the different techniques required to achieve this, we discuss its potential use in both *in vitro* and *in vivo* conditions. The proposed methodology can be used in isolation or as an adjunct to standard laboratory methods for quality control.

### The Relevance of Mitochondria to Herbal Function

Mitochondria are at the center of multicellular life. They have been known to be the site of respiration since 1949 (Kennedy and Lehninger, 1949) which already made them indispensable to most cellular functions and the very existence of multicellular life. For many years this was considered their sole responsibility with the research into mitochondria being focused on explaining the mechanisms of how this is achieved. With the discovery of the chemiosmotic gradient that drives oxidative phosphorylation (OXPHOS) to generate adenosine triphosphate (ATP) (Mitchell, 1961) their function was largely thought to be described. Recent new discoveries have demonstrated their involvement in a host of other functions necessary for multicellular life including cellular signaling, stress responses, apoptosis, cancer, ageing, inflammatory response and even mental and neurological function (Nunnari and Suomalainen, 2012; Kramer and Bressan, 2018; Annesley and Fisher, 2019). These mirror some of the main reasons why people utilise herbs (Benzie and Wachtel-Galor, 2011) leading to the possibility that mitochondria may underpin some traditional herbal concepts such as “Qi” (loosely translated as vital force or energy) which is central to the traditional descriptions of the actions of herbs (Wallace, 2008).

One of the most popular classes of herbs are adaptogens which aim to enhance our non-specific responses to stress, improving energy levels, boosting the immune system and enhancing mental functioning (Panossian and Wikman, 2010). However, their multi-target effects have made it difficult to determine any singular mechanisms by which they can be measured (Gerontakos et al., 2020). Since all of these functions can in part be attributed to mitochondria, they make an ideal group to begin analysing and profiling their effects on these organelles in order to determine the underlying mechanisms.

As well as adding to the general knowledge on these herbs, potentially providing new avenues for treatments to be developed, we suggest that mitochondria may provide a means of evaluating the quality of a herbal blend, by observing their activity on these essential organelles and comparing inter-batch variability. The strength of a system such as this is that the profile can be applied to a single herb with complex ingredients such as adaptogens, or to a blend of herbs which may also have multiple targets and mechanisms. The initial research may require some pilot work to find the ideal cell lines and specific assays to use but once profiled and recorded, future batches can be tested against the same standard. Potency can also be measured by comparing the degree to which the herb or blend creates the known effect. The advantage of using biological assays instead of chemical testing alone is that knowledge of the active ingredients or their proportion, which may be numerous and have complex interactions with each other, is not essential. Instead the effect of the herbal tea on its target tissues is measured directly and a statement of quality based on effects rather than the current methods of purported quantities of specific compounds. To discuss how this is being carried out, each specific test will be explained in turn along with its principles of operation and limitations.

## Methods of Mitochondrial Analysis

**FIGURE 2:**
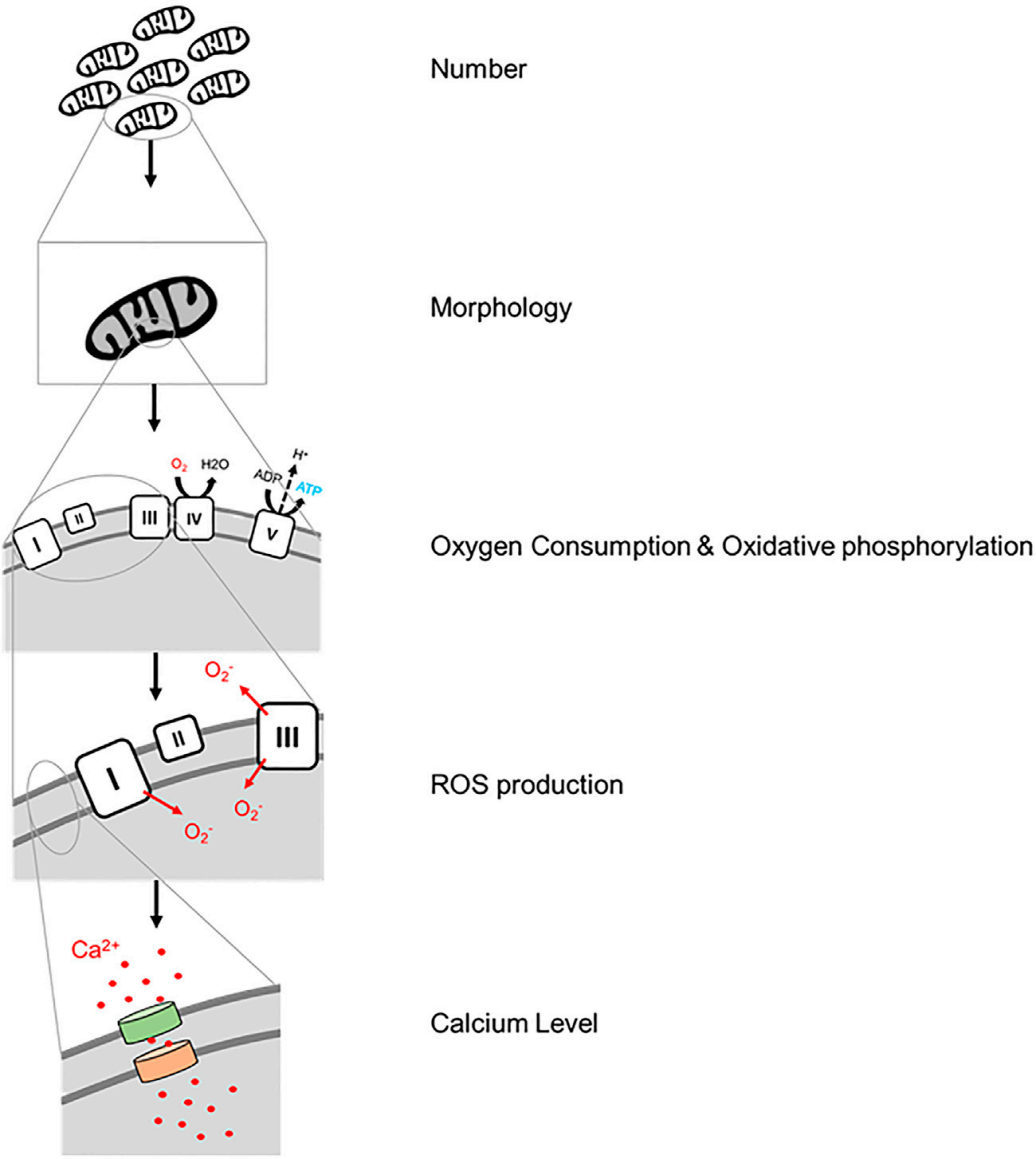
Graphical representation of the levels of mitochondrial testing that are possible today.

Mitochondrial function can be measured by a series of assays to assess their various functions. These include their quantity which reveals the balance between their biogenesis and mitophagy; their dynamic morphology into group formations; the OXPHOS cycle and its byproducts; and the signaling methods employed by mitochondria, especially their use of calcium (Figure 2).

### Mitochondrial Biogenesis and Mitophagy

Mitochondrial homeostasis is preserved by two opposing processes: biogenesis, in which new mitochondria are generated from the existing ones, and the removal of damaged mitochondria though mitophagy (Yoo and Jung, 2018; Popov, 2020). Both processes are controlled by signaling proteins that either instruct the mitochondria to self-renew, or enable autophagosomes to recognise damaged mitochondria and deliver them to lysosomes for degradation. The first step to determining the balance between these two processes is to measure the number of mitochondria present. This can be achieved by tagging the organelles with a fluorescent dye and using live cell fluorescent microscopy imaging techniques such as epifluorescence, laser-scanning confocal and spinning disk confocal microscopy, and analysing the resulting images with computer software (Viana et al., 2015). Any herbs which can be found to reliably alter the quantity of mitochondria can be assumed to be affecting their biogenesis or mitophagy. However, the number of mitochondria does not necessarily correlate with their health as disruptions in both biogenesis and mitophagy have been reported in senescence, ageing, metabolic diseases, neurodegeneration, cancer and kidney disease so further investigations into the functional capacity of the mitochondria and their behaviour in between biogenesis and mitophagy is necessary to fully understand their functioning.

### Mitochondrial Morphology and Dynamics

In contrast to the traditional depiction of mitochondria as discrete bean-shaped organelles, recent findings have revealed them to be a dynamic network that extends throughout the cell (Bereiter-Hahn and Vöth, 1994). This enables them to employ a variety of strategies to maintain their integrity under stress before becoming dysfunctional and being signaled for mitophagy. The main mechanism behind these morphological changes are the fusion and fission of their inner and outer membranes in response to metabolic stimuli. These are often related to nutrient availability and the metabolic state of the cell with nutrient withdrawal and mild stress inducing fusion to become a hyperfused network with increased OXPHOS while nutrient excess and severe stress induces fission with impaired OXPHOS and increased fragmentation (Wai and Langer, 2016). Fusion is achieved by a three step process of tethering, docking and finally fusion of their outer membranes by guanosine triphosphate (GTP) hydrolysis and may enable sharing of matrix components and protection against engulfment by phagosomes (Tilokani et al., 2018). In fission the mitochondria are encircled by a dynamin related protein (Drp1) leading to a narrowing of the membrane which is then enhanced by GTP hydrolysis marking the site of future scission. These fragmented mitochondria can then become targets for mitophagy which is important for mitochondrial quality control but excessive fragmentation and mitophagy is associated with cell death and several diseases including many neurodegenerative disorders (Pickles et al., 2018).

Mitochondrial morphology and dynamics can also be captured using imaging techniques combined with fluorescent markers and computer analysis (Harwig et al., 2018). If herbal teas can show a tendency toward fusion they may indicate increased resistance to stress, whereas a tendency toward fission will suggest increased mitophagy. In healthy cells increased mitophagy may indicate a reduced ability to adapt to stress but in damaged or diseased cells it may be part of an essential quality control mechanism. Since the quantity and morphology of mitochondria can only tell so much about the functional state of mitochondria, the next level of detail is to measure their level of functioning with their OXPHOS capacity.

### Oxidative Phosphorylation (OXPHOS)

OXPHOS is one of the main functions of the mitochondria, involving the pumping of protons into the intermembrane space and using a chain of carriers to generate an electrochemical proton gradient which is used to drive the production of ATP from adenosine diphosphate (ADP) + phosphate (P) in the presence of oxygen (O_2_) (Alberts et al., 2002). This stored energy can then be broken down again to drive the majority of reactions in eukaryotic cells. Therefore, another way of measuring mitochondrial function is to assess the concentration of ATP being generated in the cell, the mitochondrial membrane potential (ΔΨm) being generated by the proton pumps in Complexes I, III and IV of the electron transport chain (ETC), and the oxygen consumption rate (OCR) of the mitochondria.

### Adenosine Triphosphate (ATP) Synthesis

Since the final product of mitochondrial respiration is ATP synthesis, the most obvious method of assessing mitochondrial function is to measure ATP concentration. While several methods exist to measure ATP concentration (Rajendran et al., 2016), one of the most sensitive and reliable techniques is the bioluminescent luciferin–luciferase reaction (Morciano et al., 2017; Morciano et al., 2019). The enzyme luciferase, derived from the North American firefly *Photinus pyralis* L. *Lampyridae*, generates a flash of yellowish-green light proportional to the amount of ATP present as a byproduct of the oxidation of the substrate D-luciferin into oxyluciferin in the presence of magnesium ions. This light has a peak emission at 560 nm and so can be detected with luminescence detectors. Herbs which increase ATP production can be said to increase the energy reserves within our cells. The limitation of this technique is that it does not differentiate between the ATP of the cells and those from other substances such as those that may have been introduced from the herbal materials, some of which may also have their own luminescence (AIB Staff, 2013).

### Mitochondrial Membrane Potential (ΔΨm)

The ΔΨm is generated by the proton pumps of complexes I, III and IV of the ETC which create an electrochemical gradient that can be harnessed to generate ATP. High ΔΨm leads to the production of reactive oxygen species (ROS) and oxidative stress but a sustained drop in ΔΨm may also be harmful due to lack of ATP production and too low levels of ROS which may create a state of reductive stress that is just as harmful to cells as levels that are too high (Zorova et al., 2018). Herbs which increase ΔΨm may be behind ROS induced apoptotic signaling and may indicate anti-cancer effects if seen only in the cancerous cell lines and not their non-cancerous analogues. Herbs that keep ΔΨm at an optimum level, even in senescent cells, may have adaptogenic effects against ageing.

Several fluorescent lipophilic cationic dyes can be used to measure ΔΨm including:

• TMRM & TMRE (tetramethylrhodamine methyl and ethyl ester)
• Rhod123 (Rhodamine 123)
• DiOC6(3) (3,3′-dihexyloxacarbocyanine iodide)
•  JC-1 (5,5′,6,6′-tetrachloro-1,1′,3,3′-tetraethylbenzimidazolylcarbocyanine iodide)

The basic principle of all of these is the following: they accumulate within the mitochondria in inverse proportion to ΔΨm making more polarised mitochondria accumulate more dye and depolarised mitochondria accumulate less (Perry et al., 2011). This can then be detected by measuring the fluorescence or imaged with a camera. Each dye has its particular advantages and disadvantages but the overall limitations of these methods are that changes in mitochondrial morphology, localisation, or mass might also affect fluorescence measurements. Therefore controls that assess whether these changes are also happening are advisable to conduct alongside the ΔΨm probes, although these tests are also not without their limitations and may affect ΔΨm and respiration themselves Buckman et al. (2001).

### Oxygen Consumption Rate (OCR)

Oxygen plays a critical role in the generation of energy through the OXPHOS chain which is the primary source of energy in complex biological systems. Therefore, measuring the extracellular OCR in real time can reveal how much mitochondrial activity is taking place within a culture of cells. This can be measured using an extracellular flux analyser by isolating a monolayer of cells covered with an extremely small volume of media (about 2 μL) and placing a probe 200 microns above the monolayer (Agilent Technologies, 2020). The probe measures the concentrations of dissolved oxygen in the transient microchamber every 2–5 min and calculates the OCR. It then lifts allowing the media to mix with the microchamber, restoring cell values to the baseline.

Further modifications to this assay enable the determination of exactly how much oxygen is being utilised by the mitochondria for ATP synthesis, what their spare capacity is and how much is being used by other biological processes (Agilent Technologies, 2019). This is achieved by injecting up to four drugs into the sample at user specified times which affect specific complexes in the ETC. Oligomycin inhibits ATP synthase decreasing electron flow through the ETC resulting in a drop in mitochondrial respiration and OCR. By measuring the difference between normal respiration and the oligomycin adjusted OCR, the amount of O_2_ used for cellular ATP production can be calculated. Carbonyl cyanide-4 (trifluoromethoxy) phenylhydrazone (FCCP), an uncoupling agent that disrupts the ΔΨm and collapses the proton gradient, disinhibits electron flow through the ETC and causes OCR to reach its maximum. This corresponds to the ability of the cell to respond to increased energy demand under stress by enabling its spare respiratory capacity. Finally, a mixture of rotenone, a complex I inhibitor, and antimycin A, a complex III inhibitor, shuts down mitochondrial respiration completely enabling the detection of non-mitochondrial respiration by other processes such as that consumed by oxidase and oxygenase enzymes (Halliwell and Gutteridge, 2015). This can be deducted from the other measurements to acquire an accurate calculation of mitochondrial respiration. Each of these responses can then be used to acquire a detailed description of a herb’s effects.

### Reactive Oxygen Species (ROS)

Reactive Oxygen Species (ROS) are highly reactive by-products of the mitochondrial respiration process, formed when the electrons being carried by the ETC leak and combine with oxygen to form superoxide (${\text{O}}_{2}^{-}$) and/or hydrogen peroxide (H_2_O_2_), which can cause irreversible cell damage and even death (Zhao et al., 2019). Under normal physiological conditions this leak is estimated to be around 0.2–2% and serves several important functions including apoptosis, autophagy, differentiation, adaptive responses to endoplasmic reticulum (ER) stress and hypoxia, hormetic antioxidant defence and innate immunity. However, under abnormal conditions, they have been implicated in pathological states including alcohol induced and non-alcoholic liver disease, ageing, hearing loss, atherosclerosis, cardiomyopathy, ischemia/reperfusion injury, cancer, diabetes, epilepsy, Huntington’s, Alzheimer’s and Parkinson’s disease (Brand, 2016). Seeing how many of the biggest killers and causes of long term morbidity are on this list (WHO, 2018), it is not surprising that many herbal supplements and teas have based their advertising on the antioxidant effect of various compounds found in plants, despite the fact that these results have not been reproduced *in vivo* (Berger et al., 2012) and could actually be causing detrimental effects on health by affecting the regular physiological functions of ROS such as reducing the hormetic effects of exercise (Pingitore et al., 2015). Some herbs may even be useful because they increase ROS; for example, by priming cancerous cells for apoptosis making them more susceptible to chemotherapeutic agents (Henley et al., 2017).

Dichlorodihydrofluorescein diacetate (DCFDA) is one of the most widely used techniques for directly measuring the redox state of a cell (Eruslanov and Kusmartsev, 2010). It works through intracellular esterases which cleave the two ester bonds from the original molecule to produce H_2_DCF that then accumulates intracellularly and oxidises to form highly fluorescent dichlorofluorescein (DCF). This can be measured by detecting the increase in fluorescence at 530 nm when excited at 485 nm to provide a measure of generalised oxidative stress. It is unable to provide a direct measure of any particular reactive species since neither H_2_O_2_ nor ${\text{O}}_{2}^{-}$ can oxidise H_2_DCF directly but must be decomposed to radicals while other substances in the cell may also produce the reaction including other radicals as well as cytochrome c, responsible for activating the caspase cascade that initiates apoptosis, making assessment of ROS during apoptosis using DCFDA especially problematic (Lawrence et al., 2003).

MitoSOX is another assay used for detecting ROS, especially ${\text{O}}_{2}^{-}$. It contains a positively charged derivative of dihydroethidium (HE) that rapidly accumulates in mitochondria, where it is oxidised by reactive species to become 2-hydroxyethidium (2-OH-E^+^) that then binds to DNA producing a red fluorescence (Kauffman et al., 2016). This is more specific than DCFDA but it can still suffer from overlapping fluorescence from ethidium (E^+^) which is not formed from ${\text{O}}_{2}^{-}$ and may be generated in larger quantities than the radicalised form (Zielonka and Kalyanaraman, 2010; Nazarewicz et al., 2013).

Although both assays have their drawbacks, a critical approach to measuring ROS combined with data from other tests and controls can give some valuable insights into the degree of oxidative stress that a cell culture is undergoing and how exposure to herbal teas may affect this.

### Calcium (Ca^2+^) Levels

Ca^2+^ has emerged as an important signaling molecule that facilitates communication between the mitochondria and the ER enabling the mitochondria to respond to the energy demands of the cell (Rossi et al., 2019). In response to ER stress, Ca^2+^ stores are released through inositol 1,4,5-trisphosphate receptors located in numerous places where the mitochondria are in close contact with the ER. These enable the mitochondria to be exposed to far higher concentrations of Ca^2+^ than in the rest of the general cytosol where it is transported into the mitochondria by voltage-dependent anion channels located on the outer mitochondrial membrane and then taken inside the mitochondria through a channel in the inner mitochondrial membrane called the mitochondrial calcium uniporter. Moderate levels activate several enzymes of the citric acid cycle, boosting ATP synthesis and enabling mitochondrial adaptation to the cells’ metabolic needs, while high levels sensitise the mitochondria to pro-apoptotic stimuli, promoting the opening of the mitochondrial permeability transition pores, initiating cell death (Bravo-Sagua et al., 2017). This apoptotic mechanism has been implicated in several diseases including ischemia-reperfusion injury, liver and muscle diseases including cardiomyopathy, cancer, and neurodegenerative disorders (Britti et al., 2018; Romero-Garcia et al., 2019).

Ca^2+^ levels can be detected inside mitochondria using indicators like Rhod-2 acetoxymethyl (AM) ester which increases in fluorescence when it binds with Ca^2+^, excitable at 557 nm and emitting a signal at 581 nm (Cayman Chemical, 2021). This can be applied to many cell types but it is not mitochondria-specific so a second spectrally distinct dye such as MitoTracker Green may be used prior to imaging to ensure that mitochondrial calcium can be differentiated from other sources (Maxwell et al., 2018). These readings can then provide additional information on the mechanism behind increased ATP synthesis or apoptosis to determine how a herbal tea achieves an effect.

## Conclusion

Various chromatographic and spectroscopic techniques have been applied to the quality control of herbal medicines in addition to organoleptic and wet chemistry methods. Advances in analytical hardware have led to more and more detailed analysis of plant products and particularly licensed herbal medicines, which are required to conform to the standards of the national pharmacopoeias. Moreover, plant products have been shown to have a history of adulteration and contamination. Adulteration may be accidental or may result from a desire to increase profits through using cheaper ingredients. Chemical analysis methods are essential tools in the fight against poor quality and adulteration. However, although chemical purity can be readily determined, this gives little information regarding the biological activity of a given substance as herbs consist of many different compounds and it is not typically known which the active ingredient is, and current quality testing mainly relies on the analysis of marker compounds that may or may not contribute to the desired clinical effectiveness of a herbal medicine. Mitochondrial analysis presents us with a unique opportunity to address this deficiency and to develop methods that can give us a broader perspective on herbal quality and one that takes into consideration how herbs can affect biological processes. Not least for the manufacturers of such products, this offers another avenue for value addition and the means to distribute products under an entirely new marketing strategy but also for consumers, it provides the knowledge that the herbal products produced have been tested on complex biological systems and proven to be of good chemical quality.

Current analytical methods focus on determining the chemical quality of medicinal herbal teas, including adulteration and contamination. Mitochondrial analysis and associated methods propose a new way of determining the quality of plant derived products that is more closely linked to the biological activity of a product and its potential effectiveness. Future work should focus on generating data that will validate this testing methodology.
